# Time-resolved study of holeboring in realistic experimental conditions

**DOI:** 10.1038/s41467-021-27363-9

**Published:** 2021-12-01

**Authors:** J. Hornung, Y. Zobus, S. Roeder, A. Kleinschmidt, D. Bertini, M. Zepf, V. Bagnoud

**Affiliations:** 1 GSI Helmholtzzentrum für Schwerionenforschung GmbH, Planckstraße 1, 64291 Darmstadt, Germany; 2 Friedrich-Schiller-Universität Jena, Fürstengraben 1, 07743 Jena, Germany; 3 Helmholtz-Institut Jena, Fröbelstieg 3, 07743 Jena, Germany; 4 Technische Universität Darmstadt, Karolinenplatz 5, 64289 Darmstadt, Germany

**Keywords:** Laser-produced plasmas, Characterization and analytical techniques

## Abstract

The evolution of dense plasmas prior to the arrival of the peak of the laser irradiation is critical to understanding relativistic laser plasma interactions. The spectral properties of a reflected laser pulse after the interaction with a plasma can be used to gain insights about the interaction itself, whereas the effect of holeboring has a predominant role. Here we developed an analytical model, describing the non-relativistic temporal evolution of the holeboring velocity in the presence of an arbitrary overdense plasma density and laser intensity profile. We verify this using two-dimensional particle-in-cell simulations, showing a major influence on the holeboring dynamic depending on the density profile. The influence on the reflected laser pulse has been verified during an experiment at the PHELIX laser. We show that this enables the possibility to determine the sub-micrometer scale length of the preplasma by measuring the maximum holeboring velocity and acceleration during the laser-plasma interaction.

## Abstract (web summary)

Experimental measurements involving multiple laser and plasma parameters are useful in understanding the relativistic laser-plasma interactions. Here the authors extend the model of holeboring for arbitrary profiles of laser pulse and plasma scale lengths.

## Introduction

One of the outstanding questions in relativistic plasma physics is the obvious discrepancy between the kinetic energy of protons accelerated by short laser pulses predicted by theory and simulations more than 10 years ago^1–3^, and those measured in the laboratory^4,5^, so far. Despite numerous efforts both theoretically and experimentally, the gap remains, showing a clear deficiency in the understanding of the laser-plasma interaction at relativistic intensities. While many high-profile results have been published including proton energies approaching 100 MeV^6,7^, further progress requires a more detailed understanding of the interaction. For instance, the evolution of the target during the last instants, before the interaction with the peak intensity begins, requires a more thorough treatment, and subsequently one should control it to reach the ideal conditions for the desired outcome.

The interaction of short, intense laser pulses with sub-micrometer-thick targets is a field that has been numerically explored with particle-in-cell (PIC) codes extensively. For the thinnest targets, the experimental challenge is to achieve the optimal conditions identified in simulation results. One complication arises, because PIC codes typically simulate the interaction of a laser pulse with a solid target on timescales limited to few picoseconds and therefore cannot adequately describe the evolution of the target in the rising edge of the pulse on the picosecond to nanosecond scale. This requires the initial plasma parameters to be treated as a simulation input. This ad-hoc treatment is the response to the temporal contrast of the laser, that causes the light intensity to reach the ionization threshold at the target surface tens of picoseconds to a few nanoseconds ahead of the pulse peak, and to trigger an uncontrolled preplasma expansion. This uncertainty of the input state has long been considered one of the key reasons for the discrepancy between the predictions given by the PIC codes and the experimental observations^8^.

To benchmark existing PIC simulations with experiments, a precise monitoring of plasma properties like its scale length, temperature and density distribution is necessary. Interferometry or shadowgraphy were used successfully in many experiments before to characterize the preplasma expansion. Their drawback is given by the limited spatial resolution in the micrometer range^9,10^, only allowing for the measurements of long-scale preplasmas. By contrast, short scale lengths can be measured by frequency domain interferometry, when using a probe laser pulses with a duration in the tens-of-femtosecond range^11,12^. However, the necessity for a short-pulse probe beam restricts this method from being used at facilities with an intermediate pulse duration in the hundreds of femtoseconds.

A non-invasive method to gather information about the laser-plasma interaction, performed by Kalashnikov et al.^13^ in 1994 and Zepf et al.^14^ in 1996, relies on measuring the spectral change of a laser pulse which is back-reflected from the plasma during the interaction, which used the holeboring model of Wilks et al.^15^. This method was further developed to include a time resolved measurement by Sauerbrey^16^ in 1996 and Haessner et al.^17^ in 1998, and later on, at even higher intensities, to time-resolve the onset of relativistic transparency^18^. The work by Kingham et al. was crucial for these measurements, who showed the correlation between the movement of the critical density surface and the introduced phase shift of the reflected laser pulse during the interaction^19^. To use such time-resolved information about the displacement of the critical plasma density to get insight into the properties of the preplasma, a good knowledge about the behavior of the holeboring dynamics is crucial. An important step for this has been done by Iwata et al. who recently introduced the concept of an upper plasma density limit for holeboring when a constant laser intensity is applied to a exponentially growing plasma density^20^. According to this model, the laser pulse that interacts with the critical plasma density at first induces a steepening of the plasma density profile at the interaction point. This in turns slows down the holeboring velocity and a commensurate hole boring time is analytically derived.

In this paper, we extend the previous work from Iwata et al., by showing that the dynamics of holeboring is completely dominated by what happens in the preplasma region of solid targets irradiated by short-pulse lasers. First, we derive a general formulation of the holeboring velocity for any arbitrary temporal intensity and preplasma profiles. This significantly changes the picture of the non-relativistc holeboring dynamics during the interaction of a intense laser pulse with an overdense plasma, especially in the presence of a steep density profile with scale lengths in the micrometer to sub-micrometer regime. We are able to reproduce the results from 2-D PIC simulations in terms of the maximum holeboring velocity and acceleration for the specific case of the interaction of a Gaussian laser pulse with an exponential density profile of constant scale length. The maximum holeboring acceleration and velocity shows an unambiguous correlation to the laser intensity and present preplasma scale length. To verify this behavior, we performed an experimental campaign at the PHELIX laser, in which we applied time-resolved Doppler spectroscopy on the light reflected by the target to follow the hole boring dynamics, confirming the findings. In addition, we show that one can infer the preplasma scale length from a measurement of the maximum holeboring acceleration and velocity, in a time-integrated manner.

## Results

### Holeboring in an arbitrary density profile

To improve our understanding of the holeboring dynamics, we developed an analytical description for the holeboring velocity *v*(*t*) in the presence of an arbitrary plasma density profile *n*_*e*_(*x*, *t*) at the interaction point, whose position defines the critical surface. The necessity to account for an exponential density profile has been discussed by Kemp et al.^21^ and used to calculate the position of the critical surface during the interaction, which has also been used by Iwata et al.^20^ to calculate the holeboring duration and the plasma density cutoff. Our derivation is kept more general to include an arbitrary density and laser intensity temporal profile, while assuming that the preplasma is already formed at the arrival of the laser pulse. This is obtained by solving the equation of the pressure balance *n*_*i*_*M*_*i*_*v*^2^ = cos(*θ*)*I*/*c*^22^, with a spatial and temporal dependent electron density distribution *n*_*e*_(*x*, *t*) = *Zn*_*i*_(*x*, *t*), the laser intensity *I*(*t*) and extended by the influence of the incidence angle *θ*. We assume a constant ion mass *M*_*i*_ and corresponding charge state *Z* during the interaction. Note that the charge state is the charge state that yielded the plasma expansion before the interaction begins. When the hole boring process has started, further ionization of the target ions plays a negligible role, as the hole boring models only considers a momentum transfer from the laser to the ions. It has to be noted that the effect of plasma pressure increase due to plasma heating is neglected. The further assumption that we use to solve this equation is given by the electron density, which has to fulfill the relation $\frac{d}{dt}{n}_{e}(x,t)\,=\,v(t)\frac{d{n}_{e}(x,t)}{dx}$, whereas the position of the critical density surface is given by $x(t)\,=\,\int\nolimits_{-\infty }^{t}v(\tau )d\tau$, and the assumption that the interaction starts at the critical density *n*_*c*_. With the definition of the time-varying scale length ${L}_{c}(t)\,=\,{n}_{e}(x,t){(\frac{d{n}_{e}(x,t)}{dx})}^{-1}$, the equation can be solved for the velocity of the critical surface:

1 $$v(t)=\sqrt{I(t)}\left(\int\nolimits_{-\infty }^{t}\frac{\sqrt{I(\tau )}}{2{L}_{c}(\tau )}d\tau +\sqrt{\frac{c{n}_{c}{M}_{i}}{Z\,{{\mbox{cos}}}\,(\theta )}}\right)^{-1}.$$

Equation (1) shows that a time-dependent correction term, taking into a account the history of the interaction, modulates the velocity of the critical surface, such that the velocity does not adiabatically follow the laser intensity anymore. Here, it should be noted that the plasma scale length considered in the equation is the plasma scale length at the interaction region, of the initial plasma before the interaction starts. It supposes that the plasma expands slowly compared to the characteristic time scales of the laser pulses. This equation can be further simplified when assuming an exponential density profile which implies a constant scale length *L*_*c*_(*t*) = *L*_*c*_ for the initial profile at the turning point. This results in:

2 $$v(t)=\frac{2{L}_{c}\sqrt{I(t)}}{\int\nolimits_{-\infty }^{t}\sqrt{I(\tau )}d\tau +2{L}_{c}\sqrt{\frac{c{n}_{c}{M}_{i}}{Z\,{{\mbox{cos}}}\,(\theta )}}}.$$

If the scale length of the plasma tends towards infinity, the scale length dependence vanishes and the velocity can again be described by the description of Kruer et al.^22^ for a constant plasma density. This shows that the impact on the holeboring is only given for short scale length plasmas, in the sub-micrometer regime, and is therefore not in conflict with previous models or measurements^14^.

Not only the holeboring velocity increases for increasing preplasma scale lengths and laser intensities, but also the holeboring acceleration is strongly influenced by these properties, which can simply be calculated by the temporal derivative of Equation (2).

### Numerical validation

To assess the validity of our one-dimensional model, we performed 2-D PIC simulations. The simulations are performed for an exponential density profile with a certain scale length *L*_*c*_ in the sub-micrometer range and laser intensities ranging from 10^18^ W cm^−2^ to 10^21^ W cm^−2^ with a laser pulse duration of Δ*t* = 250 fs and a linear polarization. The holeboring velocity is extracted from the simulation data via spectral analysis (Doppler spectroscopy) of the reflected pulse. A Fourier analysis of the electric field yields the instantaneous wavelength of the laser pulse, which relates to the holeboring velocity, with the assumption of negligible self-phase-modulation (SPM)^23^.

**Fig. 1:**
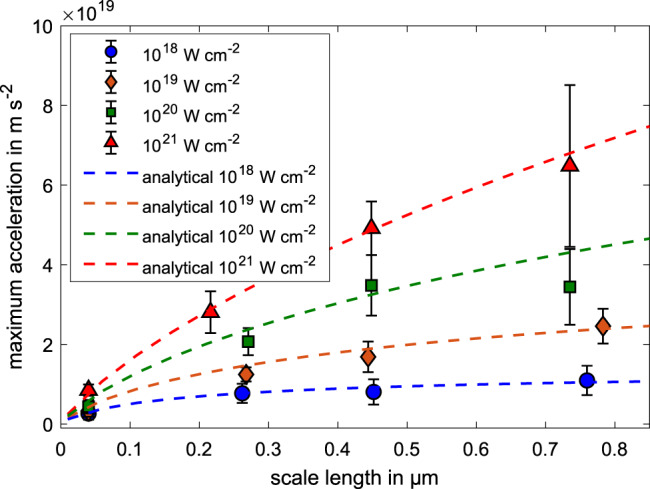
Correlation of maximum holeboring acceleration and scale length. Maximum holeboring acceleration extracted from the 2-D PIC simulation for different preplasma scale lengths and maximum laser intensities (colored shapes). The uncertainties are given by the peak-to-valley difference between different polynomial fits to the instantaneous wavelength. The dashed lines represent the calculated acceleration for Gaussian intensity distribution with a pulse duration of 250 fs, and an exponential density profile with the given scale length.

As the laser intensity rises during the interaction, the holeboring velocity and acceleration increases, which, according to the analytical description, should depend on the preplasma scale length. As a verification, we first monitored the maximum holeboring acceleration for different maximum laser intensities and preplasma scale lengths, which is visible in Fig. 1, given by the colored dots.

For a direct comparison, we calculated the maximum holeboring acceleration for a Gaussian laser intensity distribution with a pulse duration of Δ*t* = 250 fs, different peak intensities and different preplasma scale lengths, for an incidence angle of *θ* = 0^∘^. The results of the calculated maximum holeboring acceleration for different laser intensities is given by the dashed colored lines in Fig. 1, showing a very good agreement between the simulation and analytical description, even into regimes of relativistic laser intensities.

This indicates that the preplasma scale length can be retrieved using our model by measuring the maximum holeboring acceleration and the incoming laser intensity distribution.

**Fig. 2:**
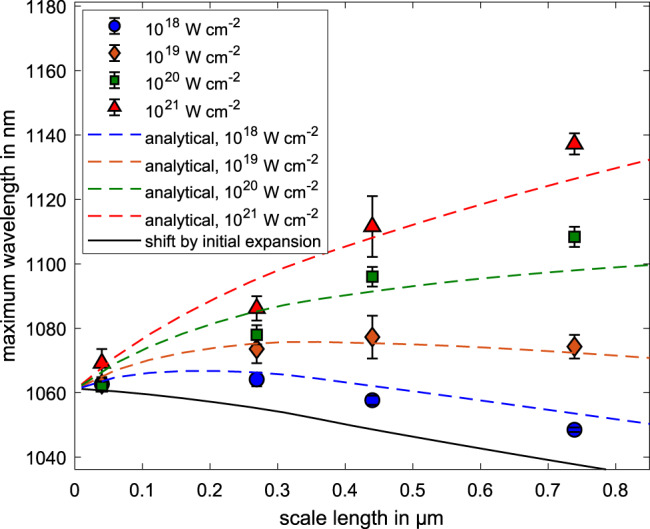
Correlation of maximum wavelength and scale length. Maximum wavelength of the reflected spectrum extracted from the 2-D PIC simulations for different preplasma scale lengths and maximum laser intensities (colored shapes). The uncertainties are given by the peak-to-valley difference between the maximum wavelength at 1 and 10% of the maximum spectral intensity. The colored dashed lines represent the maximum wavelength that has been calculated from the maximum holeboring velocity with equation (2), which is corrected by the initial shift of the preplasma expansion (black line). The initial maximum wavelength of the incoming laser pulse is given by ${\lambda }_{\max }=1061$ nm.

In addition we monitored the maximum red shift of the spectrum which corresponds to the maximum holeboring velocity, again under the assumption of negligible SPM. We extracted the maximum wavelength of the complete spectrum, averaged by the value at 1 and 10% of the maximum spectral intensity, for the different peak laser intensities and plasma scale lengths, which are shown by the colored dots in Fig. 2, with the peak-to-valley uncertainty.

In combination with this, we calculated the maximum wavelength, using the maximum holeboring velocity from Equation (2) and additionally corrected it by the initial expansion velocity of the plasma, shown by the black solid line in Fig. 2, which introduces a blue-shift offset to the spectrum. Note that the preplasma expansion was obtained by setting a non-zero start temperature in the simulation and hence plasma scale length and preplasma expansion velocity are coupled in this numerical model, as can be seen from the black solid line in Fig. 2. This shift, and therefore the initial expansion velocity could be used to determine the electron temperature of the preplasma, with the assumption of a 1-D expansion. Within the simulation a good correlation between temperature and preplasma expansion is given by the model of Samir et al. with ${v}_{{{\mbox{exp}}}}={c}_{s}\left[\right.\,{{\mbox{log}}}\,({n}_{0}/{n}_{c})-1\left]\right.$. The velocity is dependent on the initial and critical electron density *n*_0_ and *n*_*c*_ and the ion sound speed *c*_*s*_, containing the electron temperature of the plasma^24^.

Taking this initial expansion velocity into account, the dashed colored lines represent the analytical maximum wavelength of the spectrum, which again shows the same behavior as the 2-D PIC simulations. This shows that a measurement of the maximum holeboring velocity grants a possibility to determine the plasma scale length with a high precision. It is possible to directly determine these quantities with either a time-resolved or time-integrated measurement of the reflected spectrum during the laser plasma interaction.

### Measurement of the scale length at PHELIX

**Fig. 3:**
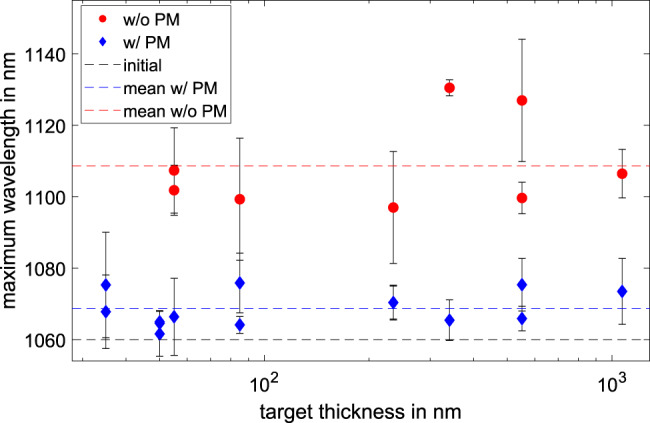
Measured maximum wavelength. Maximum wavelength of the reflected laser pulse for the case with (blue) and without (red) plasma mirrors for different target thicknesses, whereas the uncertainties are given by the peak-to-valley difference between the maximum wavelength at 1 and 10% of the maximum spectral intensity. The dashed lines correspond to the averaged values for each condition. The black dashed line shows the maximum wavelength of the incoming laser pulse.

To verify this behavior and use the derived equation to determine the preplasma scale length, we conducted an experiment at the Petawatt High-Energy Laser for Heavy-Ion eXperiments (PHELIX) at the GSI Helmholtzzentrum für Schwerionenforschung GmbH^25^, using a plasma mirror (PM) to enhance the contrast and therefore reduce the preplasma formation. The reflected spectra of the s-polarized pulse were measured and the maximum wavelengths, taken at 1 and 10%  of the spectral intensity, were extracted for different target thicknesses. The averaged results and the corresponding uncertainty (peak-to-valley) are given in Fig. 3 for the setup with and without PM, given by the blue and red points, respectively.

The spectra reflected by the target are substantially different for the two configurations, indicating that the preplasma conditions strongly influences the motion of the critical density. The spectral cutoff is shifted by (19 ± 6) nm and (52${\pm }_{16}^{17}$) nm for the case with and without plasma mirror. Additionally, it is visible that the maximum wavelength does not change when reducing the target thickness, even into regimes where a significant amount of transparency occurs, which reached up to 40 and 75% of the incoming laser energy for the case with and without plasma mirror, at a target thickness of 55 nm. This indicates that the majority of the spectral modulation occurs at the beginning of the laser plasma interaction, and is therefore dominated by the properties of the preplasma.

It is possible to use these measured shifts and the corresponding laser intensity to calculate the preplasma scale length using Equation (2), for a pulse duration of 500 fs. This results in a preplasma scale length of (0.18 ± 0.11) μm and (0.83 ± 0.39) μm for the PM and non-PM cases, respectively, with the assumption of a fully ionized plasma, without an initial expansion velocity.

## Discussion

**Fig. 4:**
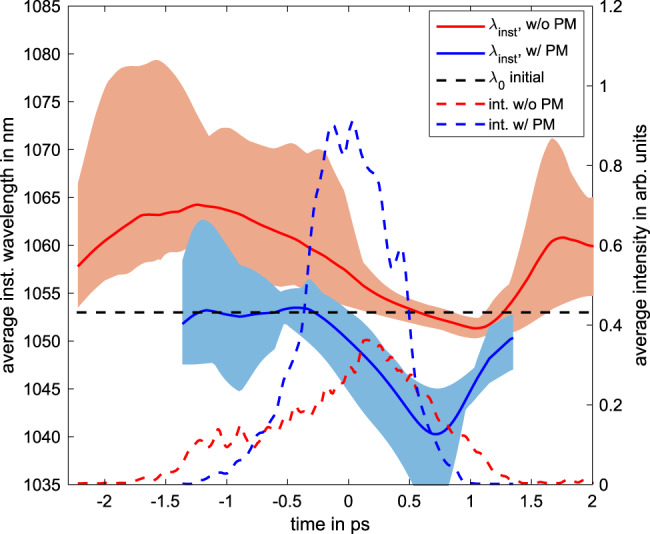
Measured instantaneous wavelength for different contrast conditions. Comparison of the instantaneous wavelengths and reflected laser pulse for the condition with (blue) and without PM (red) and an incoming laser intensity of 10^20^ W cm^−2^ and 2.4 × 10^20^ W cm^−2^, respectively. The solid blue and red lines show the mean value of all shots for each corresponding condition, with the uncertainty, given by the peak-to-valley values, indicated by the shaded areas of the respective color. The dashed blue and red lines indicate the average pulse envelope for each condition normalized to its integral and the dashed black line corresponds to the initial central wavelength.

The previous analysis has shown that the Doppler shift results from the combination of the preplasma expansion velocity and holeboring effects, which occur at different times during the interaction. A comparison with the one-dimensional model is valid if the scale length and the movement of the critical density surface is much smaller than the focal spot size. For scale lengths in the sub-micrometer regime, this requirement should be fulfilled, even for a small focal spot size of 5 μm. Therefore changing from 2-D within the simulation, to 3-D in the experimental setup should only introduce a small uncertainty. However, this has to be confirmed by 3-D PIC simulations. When the measurement is applied to the determination of the plasma scale length, the unknown charge state of the plasma has to be assumed. For the low temporal contrast, the assumption of a fully ionized plasma might be valid for the used mid-Z material, which can not be assumed for the increased contrast and therefore lower scale length. However, as Equation (2) shows, it’s influence reduces when the impact of the left-hand part of the denominator increases, which is the case for lower scale length plasma and therefore might only introduce a small uncertainty. The unknown initial expansion velocity of the plasma also introduces an uncertainty to the measurement of the scale length, which requires a time-resolved measurement for both conditions. The averaged temporally-resolved measurements of the reflected pulse are shown in Fig. 4, for the two interaction configurations, aiming at creating different preplasma expansion conditions, as measured by the frequency-resolved optical gating (FROG) technique. The instantaneous wavelength is shown in solid lines with the corresponding uncertainties given by the shaded areas. The dashed lines show the temporal envelope, which is normalized to its integral, showing a temporal broadening of the pulse in case of the low contrast.

The pulse reflected by the target is mostly blue-shifted when the PM is used due to the lower scale length of the plasma which reduces the holeboring velocity and increased counter pressure by the plasma expansion towards the end of the interaction. Additionally, there is no shift at the beginning of the interaction, showing an expansion velocity close to zero. This case shows a very good agreement with the behavior predicted by the numerical simulations. In case of the low temporal contrast, the measured pulse shows a much stronger red shift, as expected due to the increased preplasma formation, which confirms the findings detailed above. In this case however, the dynamic range is not sufficient to measure the complete interaction and the information about the initial expansion velocity is lost, which introduces an uncertainty for the scale length measurement using the maximum wavelength of the spectrum. This again shows the necessity to perform a time-resolved measurement of the reflected pulse with a sufficient dynamic range. As already mentioned, the correlation between the holeboring velocity and the critical density surface motion requires negligible SPM. Therefore, we estimated the magnitude of SPM for the mentioned parameters by considering a fast change of the electron density and therefore refractive index during the interaction, which results in an additional non-linear phase term. Even this worst-case scenario only results in very small variations of the spectrum in case of a short scale length plasma with *L*_*c*_ < *λ*_*L*_, which corroborates the work by Watts et al.^26^, showing a relevant broadening of the spectrum for *L*_*c*_ > 2*λ*_*L*_ The corresponding calculation can be found in the Supplementary Note 1, showing that it is valid to neglect the impact of SPM within the presented parameter range.

**Fig. 5:**
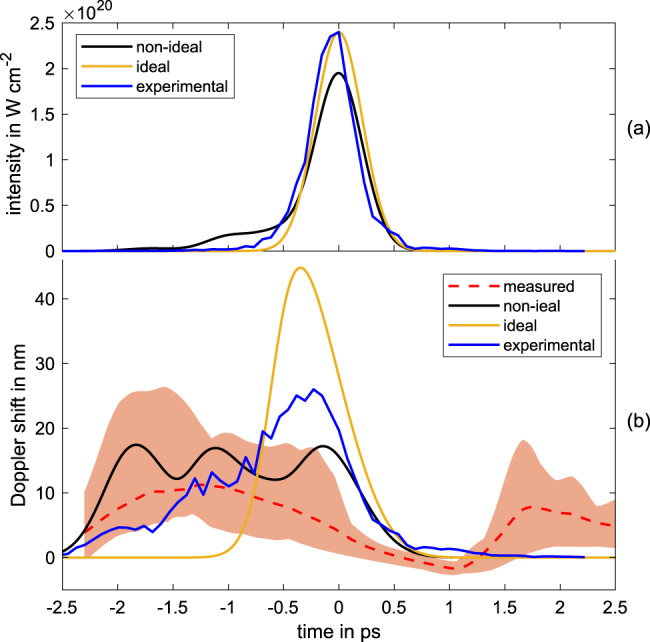
Influence of the pulse shape on the holeboring dynamic. **a** Different pulse shapes used to calculate the holeboring velocity, including an ideal Gaussian pulse (yellow), the experimentally measured incoming pulse (blue) and an arbitrary non-ideal intensity distribution (black). **b** The instantaneous wavelength, using a scale length of 0.83 μm for the different pulse shapes and the measured instantaneous wavelength, given by the dashed red line and the corresponding peak-to-valley uncertainty, given by the shaded area.

In addition, the dynamic of the phase change is slower than expected for a laser pulse with a duration of 500 fs. Therefore we calculated the expected instantaneous wavelength for different laser pulse shapes, including the incoming laser pulse that is measured during the experimental campaign, which are visible in the upper plot in Fig. 5.

The calculated instantaneous wavelength for a scale length of *L*_*c*_ = 0.83 μm, without initial expansion velocity and the different pulse shapes, for an incidence angle of 30^∘^ are shown in Fig. 5.

It is visible that a small deviation from an ideal Gaussian distribution of the incoming laser pulse shape strongly changes the instantaneous wavelength of the reflected laser pulse. This shows that the rising slope of the laser pulse has a strong impact on the holeboring and it is absolutely necessary to measure the incoming laser pulse with a sufficient dynamic range for a correct description of the holeboring evolution during the interaction. Vice versa, the measurement of the instantaneous frequency is an indicator of the on-shot laser pulse shape on a high dynamic range.

It also has to be noted that the direct correlation between the critical density movement and the phase shift of the reflected laser pulse is not necessarily given for large incidence angles and a p-polarized laser pulse^19^. For non-normal incidence, the laser pulse is reflected at an underdense layer instead of the critical density, which is given by *n*_*e*_ = *n*_*c*_cos(*θ*)^2^. However, the analytical description and correlation to the scale length holds true for the incidence angles considered here, when taking the change of the reflection point into account, which is further described in Supplementary Note 2.

This technique in combination with the derived analytical description of the holeboring velocity and acceleration seems to offer a powerful tool for the determination of the interaction dynamics in general and more particularly the preplasma properties. By extracting the acceleration from the instantaneous frequency of the reflected pulse, the maximum wavelength of the spectrum and the expansion velocity, which is given by the initial blue shift of the laser pulse, the preplasma scale length can be calculated and the temperature of the plasma estimated. This allows the determination of the preplasma properties on the picosecond time-scale before the laser peak intensity is reached, which helps to improve our knowledge about the laser-plasma interaction.

## Methods

### Experimental implementation

The experiment was conducted at the PHELIX facility. During this beamtime, the system provided intensities of up to 2.4 × 10^20^ W cm^−2^ with maximum pulse energies of 180 J on target and a pulse duration of (500 ± 75) fs using a F/1.7 focusing parabola with s-polarization. The system ensures a high temporal contrast on the nanosecond-to-100-picosecond time scale of 10^−12 ^^27^. From 100 ps on, the pulse intensity increases with a factor of 3.55 every 10 ps. The contrast was additionally improved by four orders of magnitude using a double plasma mirror (PM), which additionally steepened the rising slope of the laser pulse switch on time. The intensity after the PM dropped to ≈1 × 10^20^ W cm^−2^, at an angle between laser and target of 30 ^∘^. The used foil targets consist of polystyrene (CH) with a varying thickness between 35 nm and 1 μm.

**Fig. 6:**
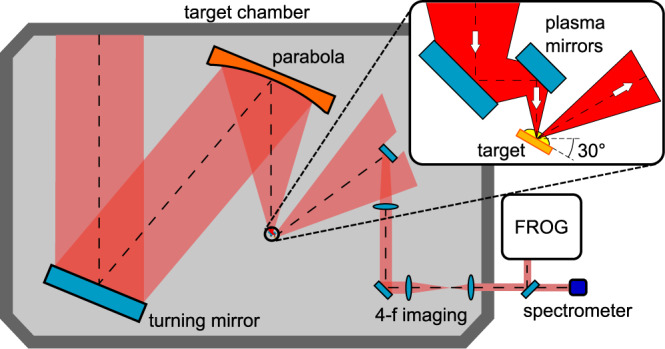
Schematic experimental setup at the PHELIX laser. The focused laser is reflected by both plasma mirrors and interacts with the solid target at an incidence angle of 30°. A part of the reflected pulse is blocked by an aperture to reduce the fluence on the following optics. Therefore only the central part of the beam is collimated and imaged onto a FROG device.

The schematic experimental setup is given in Fig. 6, showing the target chamber and beam transport to the diagnostics in addition to the setup of the double plasma mirror (inlet). The low contrast was achieved by removing the PM and realigning the focal spot to the original position, whilst maintaining the same incidence angle on the target.

A small part of the beam with a full opening angle of 3° in specular direction of the reflected beam was image-relayed to the diagnostics, which consisted of a time-resolved spectrometer based on Frequency-Resolved Optical Gating (FROG), which has been built and commissioned at our facility^28^, and a 1 *ω* time-integrating spectrometer (OceanOptics, Maya 2000-Pro) for comparison. The time-ambiguity of the SHG-FROG was overcome by a comparison to the analytical description of the holeboring velocity. This shows that the maximum red shift occurs prior to the maximum of the back-reflected intensity. This enables the possibility to flip the time of the measurements accordingly. The spectra and FROG-traces were recorded for several shots for the two different temporal contrast options and varying target thickness.

### Numerical simulations

The simulations were run with the 2-D PIC code EPOCH^29^ on the Kronos cluster at the GSI Helmholtzzentrum für Schwerionenforschung GmbH. For the simulation, a laser pulse with a central wavelength of 1053 nm, a pulse duration of Δ*t* = 250 fs (FWHM) and varying peak intensity ranging from 10^18^ to 10^21^ W cm^−2^ was incident on a solid-density target normal to the target surface, with an initial peak electron density of 4 × 10^23^ cm^−3^ and a thickness of 1 μm. The laser was focused to a spot size of 6 μm (FWHM) at the target front surface, initially located at x = 0 μm. The simulations were performed with a spatial resolution of 12 nm in the direction of the initial laser propagation, and 19.5 nm perpendicular to it. The grid size was 27,500 × 8912 cells, which corresponds to a simulation box ranging from −320 μm to 10 μm in x- and −80 μm to 80 μm in y-direction. This simulation contained ≈2.2 × 10^9^ particles. The laser pulse entered the simulation box with an intensity ratio of 10^−4^ at the simulation time *t* = 0 fs and hits the target at *t* = 1150 fs. To account for different expansion states of the target, the initial electron temperature was varied between 10 eV and 7 keV, whilst assuming a fully ionized CH_2_ target. Due to the time difference between simulation start and the arrival of the pulse at the target, a pre-expansion occurs, increasing the scale length in dependence on the initial temperature. This leads to an exponential density profile with scale lengths of 0.04 μm, 0.269 μm, 0.44 μm, and 0.739 μm for the initial electron temperatures of 10 eV, 0.5 keV, 2 keV, and 7 keV, respectively.

This overdense plasma deflects the laser towards the incoming direction, and we analyze the reflected radiation on the reflection axis. When the electromagnetic pulse propagates again in the vacuum region, an unambiguous space-time transformation is performed to obtain a temporal representation of the light pulse.

The time-resolved information is gathered by calculating the instantaneous frequency ${\omega }_{{{{{{{\rm{inst}}}}}}}}(t)=\frac{d\phi (t)}{dt}$, with *ϕ*(*t*) being the temporal phase of the pulse. The corresponding velocity of the critical density surface *v* is now calculated by solving the equation of the Doppler shift ${\lambda }_{s}={\lambda }_{0}\ \left(\right.\frac{1-2\beta \ \,{{\mbox{cos}}}\,(\theta )+{\beta }^{2}}{1-{\beta }^{2}}{\left)\right.}^{-1}$ for *β* = *v*/*c*, whereas *c* corresponds to the speed of light, *λ*_*s*_ and *λ*_0_ being the shifted and central wavelength and the incidence angle *θ*.

**Fig. 7:**
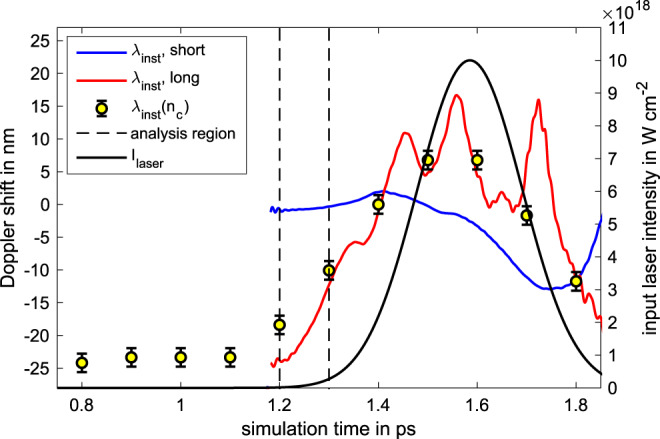
Simulated instantaneous wavelength for different scale lengths. The solid blue and red lines show the instantaneous wavelength shift of the laser pulse after the interaction with a preplasma of the scale lengths of *L*_*c*_ = 0.04 μm (blue) and *L*_*c*_ = 0.739 μm (red). As a comparison, the induced Doppler shift by the velocity of the critical density is shown by the yellow dots for the incoming temporal laser-intensity distribution, given by the black solid line. The uncertainty is given by the peak-to-valley difference of the initial expansion velocity before the laser hits the plasma. The dashed lines show the temporal region that is used to determine the holeboring acceleration.

An example for the resulting instantaneous wavelength shift of the reflected pulse with an initial intensity of 10^19^ W cm^−2^ can be seen in Fig. 7. The solid blue and red lines show the evolution of the shift for the case of a short scale length preplasma (*L*_*c*_ = 0.04 μm) and a longer scale length (*L*_*c*_ = 0.739 μm), which corresponds to a cold and hot plasma with *T*_*e*_ = 10 eV and *T*_*e*_ = 7 keV, respectively. The black solid line corresponds to the intensity of the incoming laser pulse.

In comparison, the yellow dots show the expected Doppler shift which would be introduced by the movement of the critical density surface, demonstrating a good correlation with the instantaneous Doppler shift of the reflected pulse. The shift can therefore be used as a temporally-resolved measurement of the critical-plasma-density motion during the interaction, under the assumption of negligible self-phase-modulation (SPM)^23^. The dashed black lines indicate the region that is used to calculate the maximum holeboring acceleration. The analytical holeboring solution indicates that the maximum acceleration occurs at different interaction times, depending on the laser intensity and plasma scale length. Therefore, the temporal analysis region was chosen such that the maximum acceleration should occur within these limits for all parameters. Since the instantaneous wavelength still contains some oscillations within this temporal regime, which would lead to large spikes when performing a temporal derivative, we perform polynomial fits within this temporal region, ranging from 3rd to 5th order, to get information about the averaged acceleration. The corresponding maximum acceleration values for the different polynomial orders are averaged and their respective uncertainty was included in the peak-to-valley uncertainty presented in Fig. 1.

### Theoretical background

The holeboring velocity is calculated from the balance equation between the ion momentum flux and the laser pressure.

3 $$\frac{{n}_{e}(x(t))}{Z}{M}_{i}v{(t)}^{2}=\frac{I(t)\,{{\mbox{cos}}}\,(\theta )}{c},$$

We assume a constant ion charge state *Z* and mass *M*_*i*_ during the interaction. After performing a temporal derivation of this equation it can be rearranged to a differential equation of the first degree:

4 $$v^{\prime} (t)-\frac{v(t)}{2}\frac{I^{\prime} (t)}{I(t)}+\frac{v{(t)}^{2}}{2{L}_{c}(t)}=0,$$

which accepts: $v(t)=\sqrt{I(t)}\left(\right.\int\nolimits_{-\infty }^{t}\frac{\sqrt{I(\tau )}}{2{L}_{c}(\tau )}d\tau +k{\left)\right.}^{-1}$ as analytical solution.

The integration constant *k* can be determined when assuming that the velocity of the critical density for *t* → −*∞* results in the equation (3) with a constant electron density given by *n*_*c*_, as used by Kruer et al.^22^. This results in an integration constant of $\sqrt{\frac{c{n}_{c}{M}_{i}}{Z\,{{\mbox{cos}}}\,(\theta )}}$. The introduction of a velocity offset *v*_0_ due to pre-expansion of the plasma leads to a constant velocity offset and the total velocity can be described by *v*_tot_(*t*) = *v*(*t*) + *v*_0_. For this reason a measurement of the acceleration is more favorable, since the constant term is removed during the temporal derivation. In case of a constant plasma scale length, the acceleration can be derived by the temporal derivative of Equation (2):

5 $$a(t)=\frac{\frac{{L}_{c}I^{\prime} (t)}{2\sqrt{I(t)}}\left(\int\nolimits_{-\infty }^{t}\sqrt{I(\tau )}d\tau +2{L}_{c}\sqrt{\frac{c{n}_{c}{M}_{i}}{Z\,{{\mbox{cos}}}\,(\theta )}}\,\right)-I(t)}{\left(\int\nolimits_{-\infty }^{t}\sqrt{I(\tau )}d\tau +2{L}_{c}\sqrt{\frac{c{n}_{c}{M}_{i}}{Z\,{{\mbox{cos}}}\,(\theta )}}\,\right)^{2}}.$$

This equation can be used to find the maximum acceleration during the interaction, which can be correlated to the preplasma properties. The best agreement between the simulation results and the analytical description is given when assuming a constant scale length and neglecting any temperature increase throughout the interaction. An example for this can be found in Supplementary Note 3.

## Supplementary information

Supplementary Information

## Data Availability

The experimental and simulation data that support the findings of this study are available from the corresponding author upon reasonable request. The source data underlying Figs. 1–5 and 7, as well as the Supplementary Figs. 1 and 2 are provided as a source data file. Source data are provided with this paper.

## Source data

Source data
