# Supplementary material for: Time-resolved study of holeboring in realistic experimental conditions
Source: Nat Commun. 2021 Dec 1;12:6999. doi: 10.1038/s41467-021-27363-9 (PMC8636483; doi:10.1038/s41467-021-27363-9)
Supplement: Supplementary file 1 — Supplementary Information [file 41467_2021_27363_MOESM1_ESM.pdf]

Time-resolved study of holeboring in realistic experimental conditions

J. Hornung et al.

1 Supplementary Figures

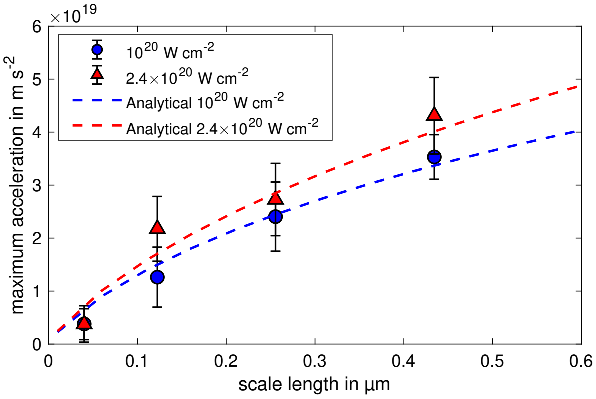

Supplementary Figure 1: **Correlation of maximum holeboring acceleration and scale length for an oblique incidence angle.** Maximum holeboring acceleration extracted from the 2-D PIC simulation for different preplasma scale lengths and maximum laser intensities (colored shapes) of $10^{20}\,\mathrm{W\,cm^{-2}}$ (blue) and $2.4{\times}10^{20}\,\mathrm{W\,cm^{-2}}$ (red), for an incidence angle of 30°. The uncertainties are given by the peak-to-valley difference between different polynomial fits to the instantaneous wavelength. The dashed lines represent the calculated acceleration for Gaussian intensity distribution with a pulse duration of 250 fs, and an exponential density profile with the given scale length.

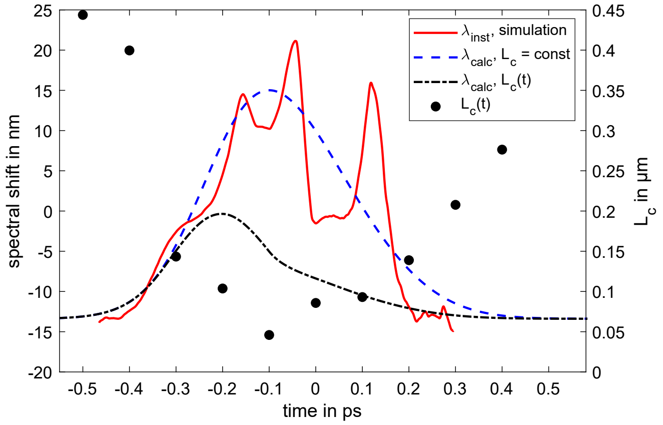

Supplementary Figure 2: **Comparison of simulated and calculated spectral shifts.** Spectral shift from the simulation (solid red) and calculated shifts using the analytical holeboring description with a constant scale length (dashed blue). The dashed black line shows the shift when using a temporally varying scale length, extracted from the simulation at the critical surface (black dots).

2 Supplementary Notes

Supplementary Note 1

Influence of Self Phase Modulation (SPM). Other spectral modulation contributions like SPM have been evaluated in order to show that they do not dominate the process. This estimate is based on a calculation of the maximum achievable optical path difference (OPD) introduced by a changing index of refraction as a function of time. SPM can be either relativistically induced or due to a fast decrease of the electron density along the beam path by hydrodynamic expansion or ponderomotive force. In all cases, this yields a fast change in index of refraction.

In the worst case, the plasma density drops such that the index of refraction reaches 1, extending up to the critical plasma density, which is the point of reflection. In such a case, the OPD over a distance X from the critical plasma density is given by:

$$\text{OPD} = X - \int_0^X \eta(x)dx \tag{S1}$$

In case of an exponentially decreasing plasma with the scale length $L(x) = n_e(x)(\frac{dn_e(x)}{dx})^{-1}$, the density can be described by $n_e(x) = n_c\exp(-\frac{x}{L})$, whereas the critical plasma density n_c is reached at $x = 0$. This can be used for the definition of the refractive index:

$$\eta(x) = \sqrt{1 - \frac{n_e(x)}{n_c}}. \tag{S2}$$

Therefore, the total OPD accumulated by the light propagating up to the critical density and back equals:

$$\begin{aligned} \text{OPD} &= 2\int_0^\infty (1 - \eta(x))dx \\ &= 2\int_0^\infty \Big(1 - \sqrt{1 - \exp\big(-\frac{x}{L}\big)}\Big)dx \end{aligned} \tag{S3}$$

The primitive of the function within the integral ($f(x) = 1 - \sqrt{1 - \exp(-x/L)}$) for x $>$ 0 is given by:

$$\begin{aligned} F(x) = &L\ln(|\sqrt{1 - e^{-x/L}} - 1|) \\ &- L\ln(\sqrt{1 - e^{-x/L}} + 1) \\ &+ 2L\sqrt{1 - e^{-x/L}} + x + C, \end{aligned} \tag{S4}$$

which can be approximated for $x >> 0$. This results in a total OPD of

$$\text{OPD} = 4L(1 - \ln(2)) \approx 1.23L. \tag{S5}$$

Therefore, the maximum OPD is of the order of the scale length, which then yields a nonlinear phase term of $\Delta\varphi = 1.23\frac{w}{c}L$.
Now, the timescales of this phase modulation has to be considered. In case of relativistic self-phase modulation, the maximum phase modulation is obtained at the maximum of the pulse intensity. In case of hydrodynamic expansion due to the temperature increase or the ponderomotive force, the timescale is similar or longer than the pulse duration such that one obtains an upper boundary for the temporal phase variation:

$$\frac{d\varphi}{dt} << 1.23\frac{\omega}{c\Delta t}L < 0.2\frac{\omega\Delta\omega}{c}L \tag{S6}$$

where the second equation is obtained from the time-bandwidth product inequality of laser pulse. This expression is the correction to the laser frequency resulting from SPM. In order for it to remains smaller than the laser bandwidth itself, we derive the following condition and its simplification:

$$0.2\frac{\omega\Delta\omega}{c}L < \Delta\omega \quad \Longleftrightarrow \quad L < 0.8\lambda \tag{S7}$$

In other words, SPM should remain minimal for small scale lengths below the laser wavelength. This estimate corroborates the results from Watts et al. [1] where SPM becomes experimentally visible for L > 2λ.

Supplementary Note 2
PIC simulation of oblique incidence. The simulations presented within the manuscript were performed for an incidence angle of 0°. However, the analytical holeboring description and the correlation to the preplasma properties also holds true for an oblique incidence angle. To show this, we also performed several simulations matching the experimental setup with an incidence angle of $\theta = 30°$. Supplementary Fig. 1 shows the maximum acceleration extracted from the instantaneous wavelength of the reflected laser pulse for different laser intensities, as used in the experimental campaign, and different plasma scale lengths.
In addition, the dashed lines indicate the maximum acceleration given by the analytical holeboring model for the same laser and plasma parameter. This shows that the analytical description is also applicable for an oblique incidence angle.

Supplementary Note 3
Assumption of constant scale length. To cross-check that the assumption of a constant scale length is valid, we calculated the expected spectral shift using the analytical holeboring velocity for different scenarios and compare it to the spectral shift, extracted from the reflected laser pulse within the particle-in-cell simulation. This has been done for a laser intensity of $10^{19}\,\mathrm{W\,cm^{-2}}$ and a scale length of 0.44 µm. The corresponding instantaneous wavelength from the simulation is visible in Supplementary Fig. 2, given by the red solid line, with the temporal change of the scale length (black dots), extracted from the

simulation directly at the critical density surface. This shows that the scale length reduces at the beginning of the interaction due to ponderomotive profile steepening.
The blue dashed line corresponds to the calculated spectral shift using the analytical holeboring description in combination to a constant scale length, showing a good agreement with the simulation. The result of the spectral shift when including the temporal varying scale length, which has been interpolated with a cubic function to match the resolution of the laser field, is given by the black dotted-dashed line. This shows that the maximum red shift is much lower than the simulation indicates and therefore does not match the observed behavior. This confirms the principle, that the holeboring velocity is influenced by the gradient of the density beyond the critical surface and any profile steepening, which needs to be overcome during the interaction. Therefore the best agreement between the critical surface motion within the simulation and the analytical holeboring description is given when assuming a constant scale length throughout the interaction. However, this does not restrict the analytical description from being used with a non-exponential density profile, since we only neglect any temporal changes due to profile steepening, but not varying scale length throughout the propagation of the density profile.

3 Supplementary References

[1] I. Watts, M. Zepf, E. L. Clark, M. Tatarakis, K. Krushel-nick, A. E. Dangor, R. Allott, R. J. Clarke, D. Neely, and P. A. Norreys, Measurements of relativistic self-phase-modulation in plasma, Phys. Rev. E, 66, 036409 (2002).
