# Supplementary material for: Time-resolved study of holeboring in realistic experimental conditions
Source: Nat Commun. 2021 Dec 1;12:6999. doi: 10.1038/s41467-021-27363-9 (PMC8636483; doi:10.1038/s41467-021-27363-9)
Supplement: Supplementary file 2 — Source data [file 41467_2021_27363_MOESM2_ESM.zip › Data description.pdf]

Data description ("Time-resolved study of holeboring in realistic experimental conditions")

The corresponding files for each figure contain the data in ASCII format. If there are any further questions regarding the dataset, please contact J. Hornung (j.hornung@gsi.de) for further information.

Figure 1:

- Acceleration taken from a polynomial fit of the instantaneous wavelength for the given laser intensity ("1e18.dat", "1e19.dat", "1e20.dat", "1e21.dat") and for different plasma scale lengths. The different files contain the scale length in µm, the acceleration in m/s^2 and the corresponding uncertainty of the acceleration in m/s^2.
- The calculated maximum acceleration can be obtained from equation (5) using a Gaussian intensity distribution with a duration of 250 fs (FWHM), an ion mass of $M = 14\ m_p$ (proton mass) and a charge state of Z = 8, R =1, an incidence angle of 0° and the critical density for a laser pulse wavelength of 1053 nm.

Figure 2:

- Maximum wavelength taken from the spectrum of the reflected laser pulse for the given laser intensity ("1e18.dat", "1e19.dat", "1e20.dat", "1e21.dat") and for different plasma scale lengths. The different files contain the scale length in µm, the maximum wavelength in nm and the corresponding uncertainty in nm.
- The calculated maximum wavelength can be obtained by calculating the holeboring velocity using equation (2), for the same parameter as described for figure 1, and by reducing this holeboring velocity by the initial expansion velocity of the plasma.
- This velocity can be calculated using the described equation by Samir, et al. for an initial temperature of 10 eV, 0.5 keV, 2 keV and 7 keV, which corresponds to the scale lengths of 0.04 µm, 0.269 µm, 0.44 µm and 0.739 µm.
- The resulting velocity can be converted to a wavelength shift using the described equation for the Doppler-shift, which is added to the initial maximum wavelength of the incoming laser pulse @ 1061 nm.

Figure 3:

- The files for the experimental maximum wavelength contain the target thickness in nm, the maximum wavelength in nm and the uncertainty of the maximum wavelength in nm, for the two cases with plasma mirror ("with-pm.dat") and without plasma mirror ("without-pm.dat").
- The mean values are calculated from the averaged maximum wavelength over all data points for each condition.

Figure 4:

- The files for the experimentally measured and averaged instantaneous wavelength with plasma mirror ("pm.dat") and without plasma mirror ("no-pm.dat"). The files contain the time relative to the first moment of the intensity distribution in seconds, the intensity in arb. units, the averaged instantaneous wavelength in m, the upper limit of the instantaneous wavelength in m and the lower limit of the instantaneous wavelength in m, which define the peak and valley of all measurements.

Figure 5:

- The files contain the time in ps, the instantaneous Doppler shift in nm, calculated using the temporal intensity distribution of the corresponding pulse in W/cm^2, for the non-ideal (non-ideal.dat), ideal (“ideal.dat”) and experimental-like (“experimental.dat”) pulse.
- The data for the measured Doppler-shift is the same as given by the file “no-pm.dat” of figure 4, from which the initial central wavelength of 1053 nm is subtracted.

Figure 7:

- The files contain the instantaneous wavelength shifts of the reflected laser pulse from the particle-in-cell simulation, for different scale lengths with 0.04 µm (“short-scale-length.dat”) and 0.739 µm (“long-scale-length.dat”). These contain the simulation time in ps and the instantaneous wavelength in nm.
- The file “critical-surface-shift.dat” contains the simulation time in ps, the shift that is introduced by the critical surface velocity in nm and the corresponding uncertainty of the shift in nm.
- The file “laser-intensity.dat” contains the simulation time in ps and the incoming laser pulse intensity in W/cm^2, which corresponds to both datasets.

Supplementary Figure 1:

- The files contain the acceleration taken from a polynomial fit of the instantaneous wavelength for the given laser intensity (“1e20.dat”, “2.4e20.dat”) of different plasma scale lengths at an incidence angle of 30°. The different files contain the scale length in µm, the acceleration in m/s^2 and the corresponding uncertainty in m/s^2
- The calculated maximum acceleration can be obtained from equation (5) using a Gaussian intensity distribution with a duration of 250 fs (FWHM), an ion mass of $M = 14\ m_p$ (proton mass) and a charge state of $Z = 8$, $R = 1$, an incidence angle of 30° and the critical density for a laser pulse wavelength of 1053 nm.

Supplementary Figure 2:

- The “inst-wavelength” files contain the relative time in ps and the instantaneous Doppler shift in nm directly taken from the simulation (“inst-wavelength-simulation.dat”), calculated with the assumption of a constant scale length (“inst-wavelength-const.dat”) and calculated with a varying scale length during the interaction (“inst-wavelength-variable.dat”) using equation (1).
- The temporally varying scale length, measured at the interaction point, is given in “time-varying-scale-length.dat”, which contains the relative time in ps and the scale length in µm. This data has been spline-interpolated to the time points of “inst-wavelength-simulation.dat” and used for the corresponding calculation of the instantaneous wavelength.
